# Exploring Spatial Relationship between Restoration Suitability and Rivers for Sustainable Wetland Utilization

**DOI:** 10.3390/ijerph19138083

**Published:** 2022-07-01

**Authors:** Nan Xu, Haiyan Li, Chunyu Luo, Hongqiang Zhang, Yi Qu

**Affiliations:** 1 Key Laboratory of Heilongjiang Province for Cold-Regions Wetlands Ecology and Environment Research, Harbin University, Harbin 150086, China; xunan0451@126.com; 2 National and Local Joint Laboratory of Wetland and Ecological Conservation, Institute of Natural Resources and Ecology, Heilongjiang Academy of Sciences, Harbin 150040, China; zzdjlnd@126.com (H.L.); iamluo2002@163.com (C.L.); hongqiangtracy@163.com (H.Z.)

**Keywords:** wetlands in Sanjiang Plain, wetland restoration, suitability evaluation, spatial relationship with rivers

## Abstract

Wetlands are important ecosystems for biodiversity preservation and environmental regulation. However, the integrity of wetland ecosystems has been seriously compromised and damaged due to the reckless and indiscriminate exploitation of wetland resources during economic development by human society. Hence, wetland restoration has now attracted wide attention. Understanding wetland restoration suitability and its relationship with river grade and river distance is an important step in further implementing wetland restoration and ensuring an orderly wetland development and utilization. In this study, wetland restoration suitability is evaluated combining natural and human factors. Taking its result as an important basis, the spatial distribution characteristics of different levels of wetland restoration suitability are discussed for the studied region; the percentage distribution of different levels of wetland restoration suitability is analyzed for 10 km long buffer zones of rivers of different grades, and the association between the distribution of different levels of wetland restoration suitability and the river distance (2, 4, 6, 8, and 10 km) is also analyzed for different buffer zones of rivers in different grades. Our findings show that the spatial distribution of wetland restoration suitability is closely associated with the grade of rivers and the distance of the wetland patches from the river. The higher the river grade, the higher the percentage of the wetland with high restoration suitability within the same river distance. The percentage of wetlands with high restoration suitability has shown a notably decreasing trend as the river distance increases for the areas beside rivers of all grades, while the percentage of a wetland area with relatively high restoration suitability tends to increase as the river distance increases for the areas beside rivers of grade I and II and does not have a noticeable trend to change as the river distance changes for the area beside rivers of other grades. Results of this can provide technical support for wetland restoration suitability evaluation for plain areas, a spatial reference for wetland restoration prioritizing, and an orderly wetland development and utilization in future studies and planning.

## 1. Introduction

Human activities have caused significant damage to wetlands [1,2]. Large-scale reduction and functional degradation of wetlands have seriously affected the sustainable development of regions [3,4,5]. Research on wetland restoration has gradually received more attention. In addition to micro-scale wetland restoration technology [6,7], research is also focused on macro-scale wetland restoration [8,9,10,11]. For example, Dale and Siobhan established a suitability model for wetland restoration using indicators such as hydric soils, land use, stream order, and saturation index [12]; Evelyn et al. combined geology, soil, topography, hydrology, drainage, and land ownership to construct a wetland restoration suitability model for wetland managers and decision-makers [13]; and Dong et al. analyzed the potential of wetland restoration from five aspects, including landscape structure, river and road density, humidity index, landform conditions, and productivity of cultivated land, in order to determine the priority of wetland restoration in the study area [14].

However, existing studies rarely consider the impact of human interference. The restoration of the wetland is a necessity due to human impact on it and thus not including the anthropogenic impacts would lead to questionable results. Different intensities of human interference, such as the duration of reclamation, the frequency of agricultural machinery use, and the total amount of fertilization, may lead to problems such as the loss of soil seed banks and soil degradation, reducing the likelihood of wetland restoration [15]. Therefore, it is necessary to incorporate human activities into wetland restoration suitability models and evaluate them through the integration of natural and human factors. This can improve the accuracy of wetland restoration assessment. In addition to providing guidelines for the reconstruction of degraded wetlands, the evaluation of wetland restoration suitability can provide spatial guidance for future wetland development. The sharp reduction of wetland area, severe fragmentation of landscape, and degradation of ecosystem services are mainly caused by development and use of wetland resources by humans, without taking into account the spatial carrying capacity [16,17]. Accordingly, it is necessary to formulate development guidelines for wetland resources. As rivers are the most prominent spatial factor, in-depth analysis of the relationship between wetland restoration suitability and stream order and buffer distances can improve understanding of the dominant spatial pattern of wetland restoration suitability and provide a reference for formulating wetland development guidelines.

The Sanjiang Plain is a globally important hotspot for wetland biodiversity, an area of ecological function at the national level, and an important base for food production in China [18,19]. In the mid-to-late 1970s, people began large-scale agricultural reclamation and construction of water conservation infrastructure, causing wetlands to decline, damaging their ecosystem and causing a series of ecological and environmental problems in the Sanjiang Plain [20,21,22,23]. Assessing the wetland restoration suitability in the Sanjiang Plain from a macro-scale perspective with the integration of natural and human factors can contribute to the determination of wetland restoration priorities. The analysis of the relationship between the spatial distribution of wetland restoration suitability and the distribution of rivers can provide a spatial reference for the future development and use of wetlands. In this study, GIS technology was applied to quantitatively assess the wetland restoration suitability in the Sanjiang Plain and its relationship with stream order and buffer distance. The aims of the study were to: (1) explore wetland restoration suitability based on the integration of natural and human factors; and (2) understand the spatial distribution of wetland restoration suitability and guide the formulation of wetland restoration, development and utilization strategies in order to effectively improve wetland restoration probability and reduce ecological loss.

## 2. Methods

### 2.1. Study Area

The Sanjiang Plain is located in northeast China with a total area of 108,900 km^2^. It is positioned between the Xiaoxing’an Mountains, the Wusuli River, the Heilong River, and Xingkai Lake. The geographical coordinates are 43°49′55″–48°27′40″ N and 129°11′20″–135°05′26″ E. The Sanjiang Plain is a freshwater swamp alluvial plain formed by the Heilong River, the Songhua River, and the Wusuli River. Due to its open and flat terrain, the Sanjiang Plain is the most important area for grain production in China. In the past few decades, the rapid development of agriculture and urbanization has negatively affected wetland resources and the overall function of wetlands in the Sanjiang Plain, resulting in a number of ecological and environmental problems such as floods, soil pollution, and water shortage. The wetland landscape is severely fragmented, causing a decline in the quality of wetland habitats and a rapid decline in biodiversity.

### 2.2. Data Sources and Processing

The fundamental data used in this study include land use data, soil data, topographic data, and yearbook statistics. Data on land use were obtained from the Resource and Environmental Science Data Center, Chinese Academy of Sciences (http://www.resdc.cn, accessed on 31 December 2012), for the period 1995–2015; data on soil type were obtained from the National Natural Science Foundation of China “Western China Environmental and Ecological Science Data Center” (http://westdc.westgis.ac.cn, accessed on 1 January 2006); a Digital Elevation Model (DEM) with 90 m resolution was extracted from the post-processed global seamless CGIAR-CSI SRTM data by CGIAR CSI and it was used as topographic data; and statistical data were extracted from the statistical yearbook of counties within the Sanjiang Plain. Saturation index, stream order and overland flow length factors used in the evaluation model were obtained through spatial analysis in ArcGIS. Hydrological soil properties were obtained by reclassifying soil types according to physical properties, while reclamation years were obtained by comparing land use maps in different years. Finally, the density of agricultural machinery and grain yield density were obtained through statistical data mapping.

### 2.3. Research Methods

#### 2.3.1. Modelling of Wetland Restoration Suitability

We used a natural and anthropogenic impact based model to assess the suitability of wetland restoration. Compared to other models, this model involved human factors into the analysis of wetland restoration suitability [24]. Indicators for the wetland restoration suitability had been selected on the basis of natural and human factors. The final list of indicators was determined in consultation with experts and literature analysis. The list was mainly based on the physical and chemical properties involved in the wetland regeneration process and human activities that affect the wetland seed bank and soil environment. Natural factors were selected from indicators that can express the physical characteristics and functions of wetland, including saturation index, soil hydrological characteristics, stream order, and overland flow length [12,25]. Human-related factors were selected from indicators that can express land use patterns and land use intensity, including land use types, reclamation years, density of agricultural machinery, and grain yield density. The saturation index and soil hydrological characteristics are the most representative physical parameters of wetlands and are key elements for wetland formation. Stream order and overland flow length are neighborhood parameters important for the formation of the specific wetland landscape [12]. These parameters can represent most of the natural factors related to the restoration of wetlands. The type and intensity of land use are the main forms of human disturbances. The land use pattern reflects the opportunity cost of wetland restoration, and the land use intensity reflects the implementation cost of wetland restoration, both of which determine the probability and complexity of successful wetland restoration [26,27].

Saturation index, stream order, and overland flow length were generated using spatial analysis based on DEM data. The saturation index is obtained from the slope and the accumulated flow length, and the formula is as follows:

SI = ln (α/tan β)(1)

where SI is the saturation index (dimensionless), α is the upstream confluence area per unit contour length at any point i on the slope, and β is the slope at that point [25,28]. The overland flow length was generated using the Flow length module in ArcGIS. Based on the river network and flow direction, the module traced all grids that flow to the nearest river along the flow direction. The distance between each grid and the next downstream grid is summed in order to obtain the overland flow length of each grid. Basin range corresponding to different stream orders was obtained using cumulative flow thresholds, and the Strahler stream order classification method was applied for river classification. The rivers directly originating from the river source was classified as the 1st order; two rivers of the same order converged to form a river of the next higher river order; the order of the river converged by two rivers at different orders was the higher of the two. Hydrological characteristics of the soil were obtained by classifying the soil type map, and the soil was divided into three categories: hydrogenic soil, soil with high water content, and other soil categories. Each soil category represented the contribution of different types of soil to the wetland restoration. The land use indicator was obtained by assigning data on the types of land use, with each type being assigned according to its contribution to wetland restoration or according to the complexity of wetland restoration. The reclamation history of cultivated land was identified by analyzing the land use transfer in different periods. Density of agricultural machinery and grain yield density was obtained by examining yearbooks of counties and cities in the Sanjiang Plain, and spatial quantitative data were obtained by assigning scores to administrative boundaries.

In this study, the Principal Component Analysis (PCA) method was used, where the main concept was to extract the principal components from the factors using the PCA method, and then combine the coefficients of variables in a linear combination of principal components with the variance contribution rate of principal components to obtain the weights through reverse calculation [29]. Prior to the analysis, a Factor Analysis (FA) of the eight influencing factors was performed to confirm that the KMO value meets the test standard and that the PCA method is suitable for the data used in this study. The eight groups of variables were then imported into SPSS 15.0 software (Armonk, NY, USA) to extract the principal components. The cumulative contribution rate of variance of the six principal components exceeded 80%. The coefficients in the linear combination of the different principal components were calculated by dividing the loading number of each variable by the square root of the corresponding characteristic root; the variance contribution rate of each principal component is considered as its weight, and the index coefficient is obtained by weighting the variance contribution rate of each principal component and the coefficients in the linear combination of principal components. Finally, the index coefficients were normalized.

The wetland restoration suitability model was constructed using simple linear weighting, where the weighted sum of each influencing factor was derived in order to calculate the wetland restoration suitability index (RSI) in each planning unit of the study area. The RSI has a continuous value between 0 and 1. Lower RSI values represent a lower restoration suitability, making it more difficult to restore through the natural form. Higher RSI values indicate a higher restoration suitability, so it is more probable that the restoration will be performed through the natural form. The construction of the model was performed using the Raster calculator tool in ArcGIS. All planning units were divided into five classes using the Natural Break (Jenks) method. Level 1 represents very low suitability for wetland restoration, Level 2 represents low suitability for wetland restoration, Level 3 represents moderate suitability for wetland restoration, Level 4 represents high suitability for wetland restoration, and Level 5 represents very high suitability for wetland restoration. The spatial distribution and the proportion of different levels of wetland restoration suitability, especially the very high and high levels, were explored, and the potential for the restoration of wetlands in the Sanjiang Plain was analyzed.

#### 2.3.2. Relationship between Wetland Restoration Suitability and Stream Orders

The first step was to determine the buffer range. In order to explore the relative breakdown of rivers with different levels of wetland restoration suitability within a certain buffer distance, a reasonable buffer distance should first be determined. In general, the higher the stream order or the larger the landscape, the greater the impact on the surrounding area of the river and the better the ecosystem services perform [30,31]. Therefore, 1st order rivers were selected as the measurement reference for determining the buffer distance, so that the influence of other rivers was also covered. One hundred sample sites were randomly selected between 1st order rivers and the boundary of adjacent river basins, and the relationship between sample distances and their restoration suitability values was analyzed by drawing a scatter diagram (taking the distance as the *x*-axis and the restoration suitability as the *y*-axis). It was observed that low values of suitability tended to stabilize at a distance of 10 km. Accordingly, 10 km was chosen as a buffer distance to examine the buffer distance of rivers of different orders. The ratio of each restoration suitability level in a certain buffer area of different orders of rivers was calculated. It was an index obtained by A_i_/A_j_, where A_i_ is the distribution area of restoration suitability of rank i in the river buffer of order j and A_j_ is the total area of the river buffer of order j. This was done in order to compare differences in wetland restoration suitability among rivers of different stream orders. In particular, the relationship between very high suitability and high suitability for wetland restoration and stream order was analyzed.

#### 2.3.3. Relationship between Wetland Restoration Suitability and River Buffer Distances

The distance between the wetland to be restored and the water sources is the key factor affecting the wetland restoration. Soil characteristics and water content are largely related to the distance from the river [32,33,34]. In general, the closer it is to the river, the greater the water inflow obtained by the wetland patch, the higher the soil water content, and the greater the possibility of recovery after the wetland patch is damaged. According to the above known understanding, this study conducted a buffer analysis on wetland restoration suitability. A secondary buffer zone was generated every 2 km within the 10 km buffer zone, forming a buffer sequence of 2 km, 4 km, 6 km, 8 km, and 10 km. The ratios of different levels of wetland restoration suitability within each secondary buffer were calculated in order to analyze the trend of wetland restoration suitability at different buffer distances. The relationship between wetland restoration suitability and its distance from the river was analyzed from two aspects: (1) how suitability levels for wetland restoration change within each stream order, and (2) how wetland restoration suitability changes across different stream orders. The main purpose of analyzing the relationship between wetland restoration suitability and distance from adjacent river is to understand the law of complexity of wetland restoration at different levels of water source availability, so as to provide reference for the utilization of wetland resources in the future.

## 3. Results

### 3.1. Weight of Wetland Restoration Suitability Indicators

Weight of each influencing factor obtained by the PCA method is shown in Table 1 (adapted from Qu et al., 2018). Among them, there were three factors that contributed most to wetland restoration. They were overland flow length and soil characteristics as natural factors, and grain yield density as human factor. The weights of overland flow length and soil characteristics were 0.3063 and 0.2304, respectively, and the weight of grain yield density was 0.1539. The weights of other factors were below 0.1.

The weights of factors indicated that the possibility of wetland restoration is not only related to the natural factors, but also depends on the impact of human activities. The natural factors played a greater role in the restoration of wetlands than human factors, with the sum of the weights of natural factors being 0.67, while the sum of the weights of human factors was 0.33. Among the natural factors, overland flow length and soil characteristics were critical factors influencing wetland restoration, which indicated that the possibility of wetland restoration is closely related to distance to the nearest river channel and hydrological soil properties. The stream order and soil permeability of the corresponding river basin had a secondary role. Among the human factors, the possibility of wetland restoration had a significant relationship with grain yield density, which reflects fertilization intensity, frequency of tillage, etc. Farmland reclamation years and land use type had a secondary role, while density of agricultural machinery played a small role.

### 3.2. Wetland Restoration Suitability Levels and Spatial Distribution

**Figure 1:**
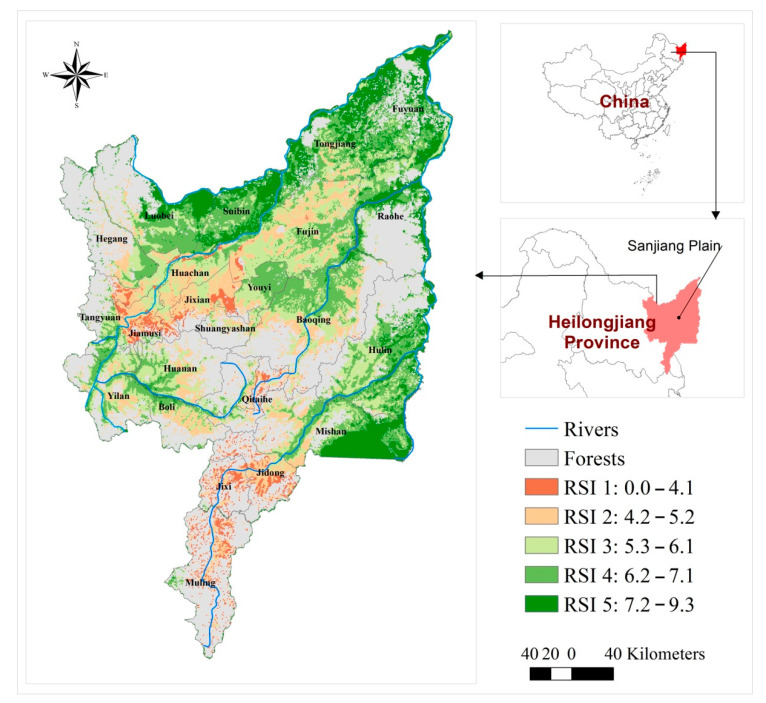
Spatial pattern of wetland restoration suitability.

The spatial distribution of wetland restoration suitability levels according to current land use is shown in Figure 1. Areas with very high and high suitability for wetland restoration were mainly distributed near Xingkai Lake and the confluences of rivers in Luobei County, Tongjiang City, Fuyuan County, Suibin County, Fujin City, Baoqing County, Mishan City, and Hulin City. These areas were not influenced by agricultural activities or had been reclaimed within 15 years (soil seed banks can still play a role in wetland restoration), so they were within the recoverable range. There was a large area of cultivated land in Fujin County that had been cultivated for less than five years, which presented a higher suitability for restoration.

Among the five RSI levels, the area with very high suitability for wetland restoration was 11,044 km^2^, which is 10.34% of the total area of the Sanjiang Plain. Combined with the area with high suitability for wetland restoration, the total area reached 33,121 km^2^, which is about 31.02% of the total area of the Sanjiang Plain. More than 1/3 of the total area had a high restoration suitability under current land use if appropriate wetland restoration was to be carried out (Table 2). The remaining wetlands in the Sanjiang Plain had an area of about 8532 km^2^ accounting for 8% of the total area, indicating that wetland partially occupied by farmlands still has a higher possibility of restoration. In Sanjiang Plain, approximately 5776.26 km^2^ of historical wetlands had been reclaimed. Spatial overlay analysis showed that 39% of the historical wetlands have very high restoration suitability and 22% of the historical wetlands have high restoration suitability. Therefore, almost 61% of historical wetlands that have been reclaimed still have the potential to be restored at a lower cost.

### 3.3. Relationship between Wetland Restoration Suitability and Stream Order

**Figure 2:**
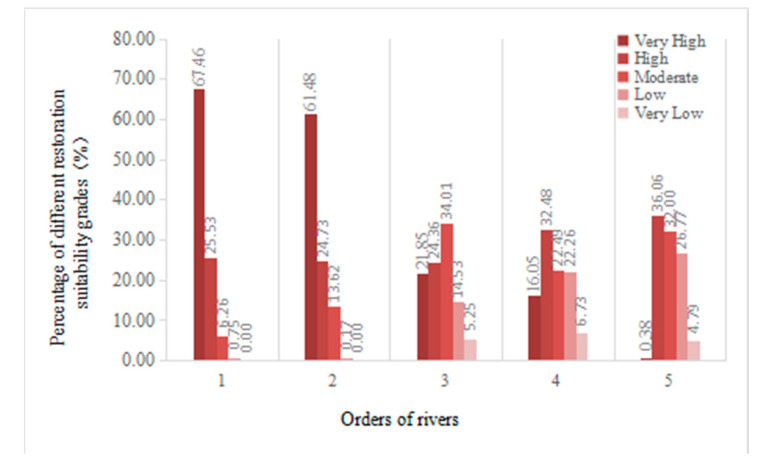
Percentage of different restoration suitability grades in the same buffer distance of different river orders.

Figure 2 shows the percentages of different levels for restoration suitability for each stream order within the same buffer distance. Overall, the 1st order and the 2nd order rivers mainly consisted of areas with very high levels for restoration suitability. The 3rd order and the 4th order rivers had a similar proportion of restoration suitability levels within a certain buffer range. In addition, they had a relatively low percentage of areas with low and very low levels for restoration suitability. The 5th order rivers had a small proportion of areas with very high and very low levels for restoration suitability within a certain buffer and mainly consisted of areas with high, moderate, and low levels for restoration suitability.

The results showed that the percentage of units with very high level for restoration suitability accounted for 67.46% of the 1st order river buffer zone, followed by 61.48% of the 2nd order river buffer zone, 21.85% of the 3rd order river buffer zone, 16.05% of the 4th order river buffer zone, and 0.38% of the 5th order river buffer zone. This showed that within a certain buffer zone, the percentage of units with very high levels for restoration suitability had a decreasing trend as the stream order decreased, and it decreased rapidly from the secondary tributaries (3rd order and 5th order) of rivers. As for the areas with a high level for restoration suitability, the percentages were almost the same in the 1st, 2nd and 3rd order river buffer zones, while the percentages in the 4th order and 5th order river buffer zones were slightly increased. The percentage of areas with moderate level for restoration suitability was relatively high in the 3rd, 4th and 5th order river buffer zones, ranging from 22.49% to 34.01%. The percentage of areas with low level for restoration suitability was basically 0 in the 1st and 2nd order river buffer zones, while the percentage in the 3rd, 4th and 5th order river buffer zones was between 14% and 27%. The percentage of areas with very low levels for restoration suitability was below 5% in all orders of river buffer zones.

### 3.4. Relationship between Wetland Restoration Suitability and River Buffer Distance

**Figure 3:**
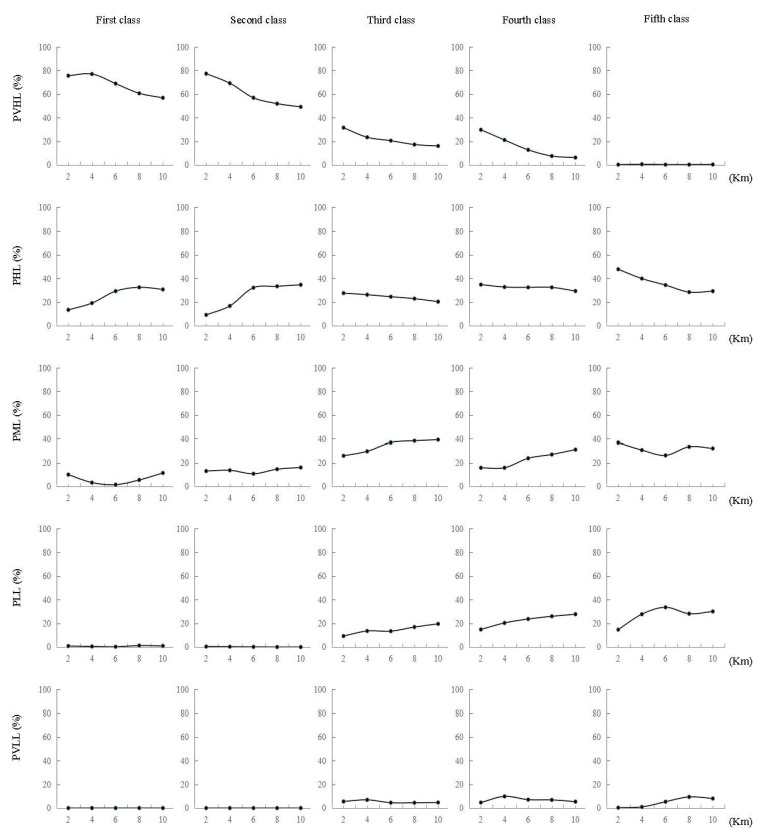
Percentage of restoration suitability in different river orders and buffer zones within different distances (PVHL means percentage of very high level; PHL means percentage of high level; PML means percentage of moderate level; PLL means percentage of low level; PVLL means percentage of very low level).

Figure 3 showed the percentages of restoration suitability levels at different buffer zone distances for each stream order. We found that the restoration suitability level has a certain association with the distance to rivers and that this association varied between rivers of different stream orders. According to the data on the very high and high levels for restoration suitability, the percentage of very high level for restoration suitability changed significantly at different buffer distances. While the very high level for restoration suitability decreased for rivers of all stream orders, the percentage in the 1st and 2nd order rivers began to decrease rapidly from a distance of 4 km, while it decreased steadily with the distance from 3rd and 4th order rivers. The percentage of high level for restoration suitability showed different changes in relation to distance to rivers of different stream orders. Its percentage gradually increased with distance and stabilized at 6 km in the 1st and 2nd order rivers, remained almost the same in the 3rd and 4th order rivers, and decreased with distance to the 5th order rivers. Although the percentage of moderate level for restoration suitability first decreased and then increased with the distance to rivers of different stream orders, overall, it showed an increasing pattern with distance. The low level for restoration suitability and very low level for restoration suitability were mainly distributed within 10 km of the 3rd, 4th and 5th order rivers, with a relatively low percentage (generally below 40%) that increased with increasing the distance to the river.

## 4. Discussion

### 4.1. Determining the Weight of Wetland Restoration Suitability Indicators

Based on the obtained results, the overland flow length and the soil hydrological characteristics were the most important natural factors, while the grain yield density was the most important human factor. In a previous study on the wetland restoration suitability, the AHP method was used to obtain the weights of influencing factors, among which the most important were land use, soil hydrological characteristics and saturation index [25]. In line with previous research, this study corroborated that soil hydrological characteristics and human factors had significant impacts on wetland restoration suitability and identified an important role of fertilization and tillage intensity. However, our findings on the importance of overland flow length and saturation index are in contrast to the results of study mentioned above [25]. This may be related to the topographic differences in the two studies. The Sanjiang Plain is flat with little variability in the saturation index and most wetlands are formed by flood pulses. As a result, the overland flow length is the dominant factor, while the impact of the saturation index is not obvious.

The results of the wetland restoration suitability model was validated by 9 long-term monitored plots for returning farmland to wetlands. About 60% of the plots restored to a degree that is equivalent to the simulated restoration suitability. However, the amount of these validation sample plots is far from enough and these plots were not widely distributed. In order to further explore the contribution of influencing factors to wetland restoration and refine the model, more restored wetland plots are needed to analyze their measured indicators at different spatial locations and with terrain different characteristics.

### 4.2. Spatial Distribution of Wetland Restoration Suitability

Wetland Restoration Suitability Index (RSI) represents the possibility of natural restoration without human assistance [12,25]. The results showed that areas with high suitability for wetland restoration in the Sanjiang Plain were mostly distributed on both sides and at the confluences of rivers of different orders. The main restoration types were cultivated land and grassland, which is in line with the study on the wetland restoration potential in eastern China [14]. There are currently eleven counties and cities in the Sanjiang Plain with a relatively high proportion of wetlands (10~20%), six counties and cities with wetlands between 5% and 10%, and two counties and cities with wetlands covering less than 5% of the area. If the wetland is not protected and restored, ecological security can be easily threatened for the continuously reduced proportion of wetland [35,36,37]. The regions with relatively high restoration suitability identified in this study can guide counties and cities with relatively low proportions of wetlands. If wetland restoration is carried out as planned, the negative effects of wetland degradation or disappearance can be significantly mitigated.

### 4.3. Relationship between Wetland Restoration Suitability and Stream Order

There is a prominent relationship between stream order and wetland restoration suitability. In general, within a certain buffer zone, the proportion of units with a high level for restoration suitability decreased with decreasing stream order. The high levels for restoration suitability levels in the 5th order river buffer zone accounted for only 0.38% of the total area, which indicates that it is difficult to develop and restore wetlands around lower order rivers. Therefore, it is necessary to avoid reclamation of wetlands near rivers below 5th order in the future development. Due to the high impact of rivers of different stream orders on wetland restoration suitability, it is necessary to further investigate the indicators that can reflect the stream order in more detail and analyze the correlation between stream order and wetland restoration suitability to formulate specific wetland restoration plans and development strategies for rivers of different stream orders. For example, NDWI (Normalized Difference Water Index) and TVDI (Temperature Vegetation Dryness Index) are important indicators for water body reflection [38,39], which can indicate the distribution of soil moisture on both river sides and reflect the stream order characteristics. Such indicators are suitable for wetlands in the Sanjiang Plain, which are mainly formed by flood pulses. Therefore, future research should consider using a remote sensing inversion index to reflect the stream order and construct corresponding quantitative indicators.

### 4.4. Relationship between Wetland Restoration Suitability and River Buffer Distance

The results on the relationship between the wetland restoration suitability and the buffer distance to the river showed that for a river of any stream order, the spatial distribution of high restoration suitability is related to the distance to the river. In general, the relationship was more obvious for rivers of higher stream order. In this study, buffer zones set at 2 km intervals could essentially reflect how the distribution of wetland suitability levels change with distance. For comparison, we can see that the proportion of low and very low wetland restoration suitability levels was almost 0 in the 1st and 2nd order rivers, but increased in the 3rd, 4th and 5th order rivers, reflecting to a certain extent the limits in distance for wetland restoration near rivers of high stream orders in plains. The moderate level for wetland restoration suitability changed with distance, i.e., it often decreased and then increased at about 6 km. Such fluctuations may be caused by a decrease in the proportion of high suitability level and an increase in the proportion of the low suitability level. The pattern of change with distance to rivers of different orders was not obvious for the moderate restoration suitability level, indicating that the moderate restoration suitability level might be affected by multiple factors. For future wetland development plans, specific distance limits should be set that correspond to rivers of different stream orders in order to resolve the difficulties in wetland restoration after the ecosystem has been damaged. 

In this study, we considered the effects of natural factors such as soil characteristics and saturation index on wetland restoration suitability of different buffer distance. However, spatial pattern of these natural factors are not permanent. With the impact of human activities, soil characteristics, saturation and other influencing factors may change in different buffer distances. For example, drainage and reclamation of wetlands will reduce soil water content, thus leading to changes in soil physical and chemical properties. Therefore, the distance limit will gradually shrink to the river direction. In addition, changes in quality of river water may also affect the possibility of wetland restoration. Here we presume that the quality of the river water is good or excellent and that it does not vary between the streams/rivers. However, the water quality of wetland is as important as the water flow in terms of ecology. The input of nutrient elements is particularly important for wetland plants. Excessive nutrient elements will restrict the growth of wetland plants and have adverse effects [40]. When the water quality changes in different rivers, the vegetation and soil quality of different buffer distance of the river will also be affected, thus affecting the possibility of wetland restoration. For future wetland development plans, specific distance limits should be set according to different stream orders and changes of physical and chemical properties in vegetation and soil, in order to resolve the difficulties in wetland restoration after the ecosystem has been damaged.

## 5. Conclusions

Combing natural and human factors, this study used remote sensing and geographic information to evaluate the wetland restoration suitability in the Sanjiang Plain from a macro perspective and analyzed the spatial relationship of restoration suitability with stream order and buffer distance from the river. The results showed that both natural and human factors had a significant impact on the wetland restoration suitability, with natural factors (sum of weights is 0.67) having a larger role than human factors (sum of weights is 0.33). Among all the factors, the overland flow length and the soil hydrological characteristics as natural factors, and the grain yield density of farmland as the human factor, are the most influencing factors. The wetland restoration suitability model established in this study can better simulate the spatial distribution of different suitability levels for wetland restoration and provide a spatial reference for wetland restoration. The results indicate that the spatial distribution of wetland restoration suitability is closely related to the stream order and the buffer distance to the river. The possibility of wetland restoration differs between the areas around rivers with different stream orders. The overall proportion of high restoration suitability in the 10 km buffer zones is higher, while the proportion high restoration suitability in different buffer strips of rivers decreases with the increasing of the distance.

Our study showed that it is feasible to simulate wetland restoration suitability from a macro perspective through the integration of natural and human factors. This approach has advantages in the analysis of the dominant spatial distribution patterns of different suitability levels. Through a macro scale simulation of wetland restoration suitability and analysis of the spatial distribution pattern of wetland restoration suitability, this study provides a reference for future wetland restoration and development strategies in the Sanjiang Plain.

## Figures and Tables

**Table 1:** Weights of factors related to wetland restoration.

Main Factors	Sub-Factors	Weights of Factors
Natural factors	(1) Saturation index	0.0509
	(2) Soil characteristic	0.2304
	(3) Stream order	0.0872
	(4) Overland flow length	0.3063
Human factors	(5) Land use	0.0876
	(6) Reclamation history	0.0819
	(7) Density of agricultural machinery	0.0017
	(8) Grain yield density	0.1539

**Table 2:** Area and proportion of wetland restoration suitability of different ranks.

Levels	Descriptions	Area (km^2^)	Percentage of the Total Area (%)
RSI 5	Very high restoration suitability	11,044	10.34
RSI 4	High restoration suitability	22,077	20.68
RSI 3	Moderate restoration suitability	18,764	17.57
RSI 2	Low restoration suitability	12,301	11.52
RSI 1	Very low restoration suitability	2908	2.72
Forests	No need for wetland restoration	39,685	37.17
